# Supplementary material for: Precise optical control of gene expression in C elegans using improved genetic code expansion and Cre recombinase
Source: eLife. 2021 Aug 5;10:e67075. doi: 10.7554/eLife.67075 (PMC8448529; doi:10.7554/eLife.67075)
Supplement: Figure 5—source data 2. — The light pulse is delivered at the 10 s mark, which corresponds to the ‘0’ mark in the graphs in Figure 5C–E and Figure 5—figure supplement 2. [file elife-67075-fig5-data2.zip › Velocities of animals in Figure 5D,E,F and figure supplement 2/Note - Figure 5C,D,E source data.rtf]

The light pulse was delivered at the 10s mark (corresponding to “0” in Figure 5C,D,E & Figure 5 - figure supplement 2)
